# The value of routine hemoglobin level evaluation following elective cesarean section: Is it worthwhile?

**DOI:** 10.1002/ijgo.15835

**Published:** 2024-08-02

**Authors:** Wisam Assaf, Maya Gruber, Eiman Shalabna, Chen Nahshon, Reuven Kedar, Ofer Lavie, Yakir Segev, Lena Sagi‐Dain

**Affiliations:** ^1^ Department of Obstetrics and Gynecology, Lady Davis Carmel Medical Center, Rappaport Faculty of Medicine Technion University Haifa Israel

**Keywords:** blood transfusion, complete blood count, cost saving, elective cesarean section, urgent cesarean section, vaginal delivery

## Abstract

**Objective:**

The aim of this study was to assess the usefulness of routine hemoglobin testing following elective and urgent cesarean section (CS) in patients without primary postpartum hemorrhage (PPH).

**Methods:**

This retrospective cohort study included women who underwent vaginal delivery (VD), elective CS, and urgent CS at Carmel Medical Center from 2015 to 2020. Data were extracted from the obstetric database, excluding deliveries with PPH. Demographic and obstetric variables were recorded. Primary outcomes were the need for packed red blood cell transfusion.

**Results:**

A total of 19 446 women were included, with five (0.3%) requiring a blood transfusion in the elective CS group, 27 (0.17%) in the VD group, and eight (0.4%) in the urgent CS group. Urgent CS was associated with a higher risk of blood transfusion, but there was no significant difference between elective CS and VD. Elective CS showed the lowest rates of post‐delivery hemoglobin below 7 g/dL 1 (0.1%) compared to VD 16 (0.6%) and urgent CS 13 (0.7%).

**Conclusion:**

Routine postoperative hemoglobin testing following elective CS in asymptomatic patients without PPH appears unnecessary. This study supports reconsidering routine hemoglobin testing following elective CS, aligning with the goal of optimizing resource utilization while maintaining patient quality.

## Synopsis

We found that routine postoperative hemoglobin testing following elective cesarean section in asymptomatic patients without postpartum hemorrhage appears unnecessary.

## 1 INTRODUCTION

The prevalence of postpartum hemorrhage (PPH) is estimated at 1%–3% of the deliveries.^1^, ^2^ Several risk factors have been identified for this phenomenon, including retained products of conception, prolonged second stage, lacerations of the birth canal, forceps or vacuum extraction, a newborn that is large for gestational age (weighing 4000 g), hypertensive disorder spectrum, and others.^3^, ^4^

The definition of PPH is variable, encompassing subjective assessments beyond standard norms, a 10% decline in hemoglobin concentration, the requirement for uterotonic medication other than prophylactic oxytocin, and the necessity for blood transfusion. For practical purposes, most obstetricians define PPH as excessive delivery‐related blood loss that leads to hemodynamic symptoms and/or hypovolemia.^5^ The most common causes of PPH are uterine atony, birth canal trauma, placenta accreta spectrum, retained products of conception, coagulopathy, and extension of the incision into the uterine vessels.^6^

The volume of blood loss during the labor process also depends on the mode of delivery. The mean blood losses for vaginal delivery (VD), cesarean delivery (CS), and cesarean hysterectomy are 500, 1000, and 1500 mL, respectively.^5^ Generally, the risks of severe maternal morbidity and mortality, including PPH, are higher in women with an urgent CS compared to an elective operation.^7^, ^8^ Thus, the rate of blood transfusion is higher among urgent CS than elective CS.^9^, ^10^

Routine postoperative hemoglobin testing on the first postoperative day (POD‐1) after CS is a widely accepted practice, as early detection of anemia can facilitate timely treatment with intravenous iron formulations and/or packed red blood cell transfusions. However, the value of routine postoperative hemoglobin testing following elective cesarean delivery in asymptomatic patients who did not have pre‐delivery anemia or develop PPH has not been widely explored.^11^, ^12^ The cost of a single test of complete blood count (CBC) is about $10, thus the aggregate costs over time may be substantial. Omission of unnecessary routine tests could result in considerable savings to the healthcare system, without adversely affecting quality of care.^11^

The purpose of this study was to assess the usefulness of routine hemoglobin testing following elective and urgent cesarean sections (CSs) in patients without primary PPH.

## 2 MATERIALS AND METHODS

This retrospective cohort study included all women who underwent VD, elective CS and urgent CS, at Carmel Medical Center during 2015–2020. The study was approved by the institutional Review Board (approval no. CMC 004‐17), and no informed consent was needed.

The common practice in the delivery room at our hospital includes routine hemoglobin evaluation before delivery for all women, as well as on the first postoperative day after every CS. Blood transfusion is suggested for any woman with a hemoglobin level below 7 g/dL, or under 8 g/dL in case of active bleeding. Typically, CBC assessment is not a standard procedure following VD, unless specific conditions are met. These conditions include instances of active bleeding during or after delivery, patients who exhibit symptoms of weakness and dizziness along with abnormal vital signs, such as tachycardia and/or hypotension, or situations where there is suspicion of infection.

Data were extracted through a computerized search of the obstetric database, which records all deliveries of any pregnant woman admitted to the hospital. Deliveries with PPH were excluded because this condition requires CBC evaluation,^13^ while the aim of this study was to evaluate hemoglobin testing in post‐deliveries without overt hemorrhage. Several demographic and obstetric variables were recorded, including maternal age, gestational age at delivery, multiple gestation, neonatal birth weight, admission platelet (PLT) count, indications for CS, pre‐delivery/preoperative and postoperative hemoglobin levels, and packed red blood cell (RBC) administration.

The primary outcomes were the need for packed RBC transfusion. For this purpose, the rate of blood transfusion between urgent and elective CS, as well as VD. Secondary outcomes included the rate of post‐delivery hemoglobin levels below 7 g/dL and the difference in pre‐ and postoperative hemoglobin.

The statistical analysis was conducted using SPSS for Windows version 28 (SPSS Inc., Chicago, IL, USA). Two‐tailed tests with a significance level of 5% were employed for all analyses. Continuous variables are expressed as means with standard deviations and were compared among the three cohorts (VD, elective CS, and urgent CS) using the Student's *t*‐test. Categorical variables are presented as numbers (percentages) and were compared using the chi‐squared test. The degree of association was quantified using odds ratios (OR) along with 95% confidence intervals (CI).

## 3 RESULTS

Overall, 19 446 women were included in the study. Of these, 15 908 (81.8%) women underwent VD, 1668 (8.6%) had elective CS, and 1870 (11.7%) urgent CS.

The demographic data of the participants are presented in Table 1. In the elective CS group, the women were older, had a lower gestational age, higher rate of multiple gestations, and higher birth weights, compared both to the VD and to urgent CS groups. Additionally, lower levels of PLT were noted in the elective CS group compared to the VD group. The indications for elective CS are shown in Table 2, the most common of these a previous CS (943 [55%]).

**TABLE 1:** Demographic characteristics of the vaginal delivery, elective and urgent cesarean section (CS) groups.

	Vaginal delivery *N* = 15 908	Elective CS *N* = 1668	Urgent CS *N* = 1870	*P* value
Maternal age	31.1 ± 4.8	33.5 ± 5.1	31.8 ± 5.2	<0.001^a^, ^b^, ^c^
Gestational age	39.4 ± 1.3	38.5 ± 1.1	39.2 ± 1.7	<0.001^a^, ^b^, ^c^
Multiple gestation	155 (1.0%)	120 (7.2%)	55 (2.9%)	<0.001^a^, ^b^, ^c^
Birth weight	3.3 ± 0.46	3.4 ± 0.71	3.3 ± 0.60	<0.001^a^, ^c^
PLT	211.5 ± 58.0	208.5 ± 58.4	210.9 ± 61.0	0.032^a^

Abbreviation: PLT, platelets.

^a^ Significance difference after Bonferroni correction between vaginal and elective.

^b^ Significance difference after Bonferroni correction between vaginal and urgent.

^c^ Significance difference after Bonferroni correction between elective and urgent.

**TABLE 2:** Indications for elective cesarean section (CS; *N* = 1668).

Indication for elective CS	*N* = 1668 (%)
Breech presentation	223 (13.4)
Multiple gestation	90 (5.4)
Failed induction	25 (3.1)
Previous one cesarean delivery	552 (31.5)
Previous multiple cesarean delivery	391 (23.5)
Maternal request	57 (3.4)
Suspected macrosomia^a^	81 (4.9)
Other indications^b^	249 (15.0)

^a^ Sonographic estimated fetal weight above 4.5 kg in low‐risk pregnancy, and above 4 kg in maternal diabetes (gestational or pre‐gestational).

^b^ Other indications include maternal active herpes infection, intrauterine growth restriction, placenta previa or accreta spectrum, previous stage 3 or 4 perineal laceration, previous shoulder dystocia, past obstetrical trauma, obstructive vaginal papilloma infection, tumor previa.

Forty women required a blood transfusion: 27 (0.17%) in the VD group, five (0.3%) in the elective CS group, and eight (0.4%) in the urgent CS group, *P* = 0.044 (Table 3). Using multivariable logistic regression (including maternal age, gestational age, multiple pregnancies, birth weight, and platelet levels), only the type of delivery showed an association with the need for blood transfusion. Compared to VD as a reference, urgent CS was found to increase the risk of postoperative blood transfusion, with an adjusted OR of 2.37 (CI: 1.07–5.25), *P* = 0.033. There was no statistically significant difference in blood transfusion between elective CS and VD—OR 1.53 (95% CI: 0.58–4.01), *P* = 0.392.

**TABLE 3:** Packed cell administration.

	Packed cell administration		*P* value
	No	Yes	
	*N* = 19 406	*N* = 40 (%)	
Vaginal delivery *N* = 15 908	15 881	27 (0.17)	0.044
Elective cesarean section (CS) *N* = 1668	1663	5 (0.3)	
Urgent CS *N* = 1870	1862	8 (0.4)	
Age (years)	31.3 ± 4.9	31.6 ± 5.3	0.708
GA (weeks)	39.3 ± 1.4	38.9 ± 1.7	0.051
Multiple gestation	329 (1.7)	1 (2.5)	0.496
Birth weight	3.3 ± 0.50	3.2 ± 0.60	0.323
PLT	211.2 ± 58.3	201.5 ± 68.8	0.248

Abbreviations: GA, gestational age; PLT, platelets.

Both elective and urgent CS groups had lower pre‐delivery hemoglobin levels compared to the VD group. Post‐delivery CBC was noted in 2780 vaginal deliveries. The mean decrease in Hb was lowest in the VD group (0.63 ± 1.0 g/dL), followed by elective CS (1.16 ± 0.86 g/dL), and urgent CS (1.52 ± 1.12 g/dL). The mean hemoglobin difference pre‐ and post‐delivery, as well as hemoglobin difference above 2 g/dL, showed statistical significance between the three groups, *P* < 0.001 for both comparisons, with the hemoglobin drop being lowest in VD. Conversely, lowest rates of post‐delivery hemoglobin below 7 g/dL were found in elective CS (1 [0.1%]), significantly lower compared to the VD group (16 [0.6%]) and to the urgent CS group (13 [0.7%]), *P* = 0.014 (Table 4).

**TABLE 4:** Hemoglobin parameters peripartum.

Delivery type/hemoglobin parameters	Vaginal delivery (*n* = 2780)	Elective cesarean section (CS) (*n* = 1641)	Urgent CS (*n* = 1831)	*P* value
Mean hemoglobin pre‐delivery	12.2 ± 1.2	10.8 ± 1.2	10.5 ± 1.8	<0.001^a^, ^b^, ^c^
Hemoglobin difference (g/dL) Mean ± SD g/dL	0.63 ± 1.0	1.16 ± 0.86	1.52 ± 1.12	<0.001^a^, ^b^, ^c^
Hemoglobin difference >2 (g/dL)	*N* = 2780 307 (11.0)	*N* = 1641 251 (15.3)	*N* = 1831 606 (33.1)	<0.001^a^, ^b^, ^c^
Hemoglobin postpartum <7 (g/dL)	*N* = 2780 16 (0.6)	*N* = 1641 1 (0.1)	*N* = 1831 13 (0.7)	0.014^a^, ^c^

^a^ Significance difference after Bonferroni correction between vaginal and elective.

^b^ Significance difference after Bonferroni correction between vaginal and urgent.

^c^ Significance difference after Bonferroni correction between elective and urgent.

## 4 DISCUSSION

In this study, no statistically significant difference was observed in blood transfusion rates between VD and elective CS in patients without evident PPH. This outcome is in line with previous studies where a greater likelihood of increased transfusion risk in cases of urgent CS was reported.^9^ This consistency supports our initial hypothesis that performing a CBC on the first postoperative day is unnecessary in elective CS.

In many obstetric departments, the routine assessment of hemoglobin levels is a customary procedure following a CS. The rationale behind advocating for this practice is to promptly detect and address early anemia, with the aim of enhancing maternal well‐being. However, the necessity of this practice remains unproven. A similar study conducted by Horowitz et al. involving 383 patients came to the conclusion that routine postoperative hemoglobin testing after an uncomplicated elective CS is unnecessary and should be omitted in asymptomatic, low‐risk women.^11^ Likewise, Api et al. showed that routine hemoglobin testing after an uneventful unplanned CS in 2450 women neither modified postoperative management nor identified the patients requiring blood transfusions.^12^

Previous studies have indicated postoperative blood transfusion rate ranging from 1.1% to 7.8% in developed countries, and up to 25% in developing countries.^10^, ^14^, ^15^, ^16^ In the current study, the blood transfusion rate within the elective CS group was 0.3%, which is lower than the 1.3% rate reported by McMahon and colleagues in cases of elective CS due to the indication of previous prior CS or following an unsuccessful trial of labor.^17^ However, it is important to note that we excluded deliveries with overt PPH, which could account for the observed variance. In conjunction with our results, in a study conducted by Chua et al. the blood transfusion rate was reported as 0.39% in elective CS.^9^

Several studies evaluated the cost‐effectiveness of routine type and screening testing for expected VD and for CS, and concluded that this screening does not seem to enhance patient care, and should be eliminated for patients without substantial risk factors,^14^, ^18^ but this is beyond the scope of this study.

Interestingly, the lowest rates of hemoglobin levels below 7 g/dL were observed in elective CS (0.1%), when compared to VD (0.6%) and urgent CS (0.7%). As mentioned earlier, CBC after VD was performed in high‐risk patients and was not a routine procedure for all vaginal deliveries (see Section 2), which may have had an impact on the results.

The rising costs in today's healthcare, combined with decreasing reimbursements from third‐party sources, compel healthcare providers to assess the necessity of routine laboratory tests. Even if the individual test costs are modest, the cumulative expense over time can be significant. Eliminating unnecessary routine tests has the potential to generate substantial savings for the healthcare system without compromising the quality of care. A reduction in routine hemoglobin testing after elective CS would result in considerable cost savings. In the authors' country, the cost of a single CBC test is approximately $10, and it takes about 5 min to be processed. Since in Israel, about 21 300 elective CS are performed each year,^19^ the annual cost of universal testing of CBC alone for the healthcare system is estimated at $213 000. According to our findings, the potential financial savings due to eliminating these procedures would not affect the quality of care. Further cost effectiveness prospective study should be done.

The strength of this study lies in its incorporation of a large number of participants, as well as the exclusion of patients with PPH for whom a CBC is justified. A limitation of the study was the lack of CBC data after uncomplicated or asymptomatic VD, which is crucial for comparison with uneventful elective CS. Another limitation was the absence of a correlation between blood transfusion and the indication for CS. Additionally, the study was constrained by the low number of instances of blood transfusion. Moreover, information about intravenous iron administration was missing, and there were no clear criteria for its administration.

Another limitation of the study was that various factors contributed to the complexity of the analysis, such as the differences between elective CS and VD patients, the higher blood loss associated with CS deliveries, variations in post‐delivery CBC practices, lack of specific reasons for transfusion in asymptomatic patients, and the absence of a formal cost‐effective analysis. For more conclusive results, a randomized controlled trial comparing elective CS patients with and without routine day‐1 CBC alongside a detailed cost‐effectiveness evaluation is recommended.

Clinical pathways have become a common practice in numerous obstetric institutions, with their primary objectives being the maintenance or enhancement of quality while minimizing unnecessary resource utilization. Typically, these pathways are formulated through a combination of literature review and expert consensus to establish an optimal care plan for uncomplicated patients. However, existing literature does not thoroughly explore the cost‐effectiveness of CBC elective CS, and as a result, many clinical pathways continue to include this test based on tradition. To ascertain the necessity and potential to enhance patient quality, further cost analysis is imperative for all “routine” laboratory testing. Regarding routine testing for patients admitted for an elective CS, our analysis failed to present compelling evidence that this common test significantly improves patient quality. Therefore, it is advisable to eliminate it from standard clinical pathways for patients PPH.

## AUTHOR CONTRIBUTIONS

**Wisam Assaf**: Project development, data collection, data analysis and manuscript writing. **Maya Gruber**: Data collection. **Chen Nahshon**: Data collection. **Eiman Shalabna**: Data analysis. **Reuven Kedar**: Data collection and data analysis. **Ofer Lavie**: Data analysis and manuscript writing. **Yakir Segev**: Data analysis and manuscript writing. **Lena Sagi‐Dain**: Project development, data analysis and manuscript writing.

## FUNDING INFORMATION

No funding was received for this study or for its publication.

## CONFLICT OF INTEREST STATEMENT

The authors have no conflicts of interest.

## Data Availability

Research data are not shared.
